# SARS-CoV-2-specific immune responses converge in kidney disease patients and controls with hybrid immunity

**DOI:** 10.1038/s41541-024-00886-0

**Published:** 2024-05-28

**Authors:** Muriel Aguilar-Bretones, Yvette den Hartog, Laura L. A. van Dijk, S. Reshwan K. Malahe, Marjolein Dieterich, Héctor Tejeda Mora, Yvonne M. Mueller, Marion P. G. Koopmans, Marlies E. J. Reinders, Carla C. Baan, Gijsbert P. van Nierop, Rory D. de Vries, RECOVAC Consortium, Alferso C. Abrahams, Marije C. Baas, Marc H. Hemmelder, Pim Bouwmans, Marc A. G. J. ten Dam, Lennert Gommers, Aiko P. J. de Vries

**Affiliations:** 1 Department of Viroscience, Erasmus Medical Center, Rotterdam, The Netherlands; 2 Department of Internal Medicine, Nephrology and Transplantation, Erasmus Medical Center Transplant Institute, Rotterdam, The Netherlands; 3 Department of Immunology, Erasmus Medical Center, Rotterdam, The Netherlands; 4 Department of Nephrology and Hypertension, University Medical Center Utrecht, Utrecht, The Netherlands; 5 Department of Nephrology, Radboud University Medical Center, Radboud Institute for Health Sciences, Nijmegen, The Netherlands; 6 Division of Nephrology, Department of Internal Medicine, Maastricht University Medical Center and CARIM School for Cardiovascular Disease, University of Maastricht, Maastricht, The Netherlands; 7 Dutch Registry RENINE, Nefrovisie, Utrecht, The Netherlands; 8 Department of Nephrology, Leiden University Medical Center, Leiden, The Netherlands

**Keywords:** Outcomes research, Preclinical research

## Abstract

Healthy individuals with hybrid immunity, due to a SARS-CoV-2 infection prior to first vaccination, have stronger immune responses compared to those who were exclusively vaccinated. However, little is known about the characteristics of antibody, B- and T-cell responses in kidney disease patients with hybrid immunity. Here, we explored differences between kidney disease patients and controls with hybrid immunity after asymptomatic or mild coronavirus disease-2019 (COVID-19). We studied the kinetics, magnitude, breadth and phenotype of SARS-CoV-2-specific immune responses against primary mRNA-1273 vaccination in patients with chronic kidney disease or on dialysis, kidney transplant recipients, and controls with hybrid immunity. Although vaccination alone is less immunogenic in kidney disease patients, mRNA-1273 induced a robust immune response in patients with prior SARS-CoV-2 infection. In contrast, kidney disease patients with hybrid immunity develop SARS-CoV-2 antibody, B- and T-cell responses that are equally strong or stronger than controls. Phenotypic analysis showed that Spike (S)-specific B-cells varied between groups in lymph node-homing and memory phenotypes, yet S-specific T-cell responses were phenotypically consistent across groups. The heterogeneity amongst immune responses in hybrid immune kidney patients warrants further studies in larger cohorts to unravel markers of long-term protection that can be used for the design of targeted vaccine regimens.

## Introduction

Kidney disease patients are at risk for more severe coronavirus disease 2019 (COVID-19) compared to the general population. This was especially pronounced in the initial waves prior to availability of vaccines and could be attributed to underlying comorbidities and a uremia- and drug-induced chronic immunosuppressive state^1–3^. As mRNA-based COVID-19 vaccines became available, these were directly offered to kidney disease patients, and shown to induce humoral and cellular immune responses in kidney patients on dialysis and in patients with stage 4/5 chronic kidney disease (CKD)^3–5^. However, lower Spike (S)-specific binding and neutralizing antibody levels compared to those in healthy individuals were observed after vaccination^3–5^. In comparison to controls, kidney transplant recipients (KTR) had severely impaired or even undetectable vaccine-induced immune responses post-vaccination^3–9^.

In the beginning of the COVID-19 pandemic, prior to the marketing of vaccines, SARS-CoV-2-specific immune responses could exclusively be acquired by infection. Later, mass vaccination campaigns led to vaccine-induced immune responses in the majority of the population. During these vaccination campaigns, individuals with a prior SARS-CoV-2 infection were also vaccinated, leading to ‘hybrid immunity’; immunity acquired by a combination of a SARS-CoV-2 infection followed by COVID-19 vaccination. When antigenically distinct SARS-CoV-2 variants emerged^10^, especially those from the Omicron sub-lineage, vaccine breakthrough infections became common, which led to hybrid immunity acquired by infection after vaccination in a large proportion of the population. A recent review and meta-regression analysis showed that hybrid immunity can provide superior protection against COVID-19^11–13^. It was shown that vaccination of COVID-19 convalescent individuals elicited broader and more potent cellular and humoral immune responses compared to vaccination of immunologically naive individuals, potentially explaining this superior protection^14–19^. As for COVID-19 convalescent kidney disease patients, mRNA-based COVID-19 vaccination also led to more robust humoral and cellular immune responses as observed in naive vaccinated patients^20–24^. However, the kinetics of antibody responses and the phenotypic characteristics of SARS-CoV-2 specific B- and T-cell responses in hybrid kidney disease patients are poorly defined.

In this study, we investigated longitudinal SARS-CoV-2-specific immune responses post-vaccination in a small cohort of kidney patients with hybrid immunity from a multicenter cohort vaccination study: the REnal patients COVID-19 VACcination Immune Response (RECOVAC IR)^3^. We focused on kidney patients with CKD G4/5, patients on dialysis, and KTR, who had recovered from prior asymptomatic or mild COVID-19 and subsequently received two doses of the COVID-19 mRNA-1273 vaccine (Hybrids) and those that were vaccinated only (vaccinees). We measured SARS-CoV-2 S-specific binding and neutralizing antibodies towards the ancestral strain and the Alpha, Delta and Omicron BA.1 variants. Additionally, we measured the frequency and studied the phenotype of S-specific B- and T-cells before and after vaccination in hybrid kidney disease groups and controls. We show that hybrid immunity in kidney disease patients is characterized by robust S-specific B- and T-cell immune responses in kidney disease patients that are of similar or even bigger magnitude compared to hybrid controls. This study provides in-depth insights into the characteristics of hybrid immune responses in individuals with kidney disease compared to controls that aid in the design of targeted vaccine regimens.

## Results

### Cohort characteristics

Initial participants of the RECOVAC IR study that were later excluded because of evidence of SARS-CoV-2 infection prior to vaccination were included in this hybrid immunity study. All patients had asymptomatic or mild COVID-19. We analyzed 6 individuals with no kidney disease (controls), 6 patients with CKD G4/5 (CKD), 9 patients on dialysis, and 3 KTR. For serological analysis equal numbers of vaccine recipients without hybrid immunity were included (Table 1). The median estimated glomerular filtration rate (eGFR) for hybrid controls, CKD G4/5 patients and KTRs was 72.2 (55.7–125.3), 9.2 (7.5-21.2) and 58.2 (31.2–62.1) mL/min/1.73m2, respectively. Among KTRs, the median time since their last transplantation was 9 years (4-19) for hybrids and 6 years (2–7) for vaccinees. The median age of hybrid controls (57.5 years) was lower than that of other hybrid groups (63 years). Among vaccinated individuals, the median age of KTRs (47.0 years), was lower than other groups (60 years). As expected, inclusions with hybrid immunity had S1- and N-specific antibodies at baseline, while naïve individuals were seronegative for both. S1- and N-specific IgG levels varied among the hybrid groups, with the highest levels in dialysis and the lowest in KTR.

**Table 1:** Baseline characteristics of participants with hybrid SARS-CoV-2 and vaccine induced immunity

Study group	Hybrid immune (*n* = 24)				Vaccine immune (*n* = 24)			
	Control	CKD	Dialysis	KTR	Control	CKD	Dialysis	KTR
Number of participants, n (M:F)	6 (4:2)	6 (5:1)	9 (5:4)	3 (2:1)	6 (3:3)	6 (5:1)	9 (7:2)	3 (1:2)
Age, yr (range)	58 (22–76)	70 (62–77)	63 (39–68)	61 (24–78)	57 (39–66)	64 (21–82)	69 (48–78)	47 (37–61)
BMI, kg/m^2^ (range)^a^	30 (25–33)	30 (25–33)	27 (22–38)	25 (21–28)	29 (25–37)	27 (23–32)	24 (20–33)	34 (31–35)
Lymphocytes,10^9^cell/L (range)	2.1 (1.2–2.3)	1.5 (0.8–2.4)	1.2 (0.7–1.9)	2.4 (1.0–2.4)	2.6 (1.5–3.7)	1.6 (1.0–2.1)	1.4 (0.9–2.4)	3.7 (2.5–4.9)
eGFR, mL/min/1.73m^2^ (range)	73 (56–125)	9 (8–21)	—	58 (31–62)	81 (61–111)	15 (11–21)	—	49 (33–64)
Dialysis characteristics								
Hemodialysis, *n* (%)	—	—	7 (78%)	—	—	—	7 (78)	—
Peritoneal dialysis, *n* (%)	—	—	2 (22%)	—	—	—	2 (22)	—
Months on dialysis, *n* (range)	—	—	27 (10–481)	—	—	—	39 (1–379)	—
Transplant characteristics								
Yr post-transplantation (range)	—	—	—	9 (4–19)	—	—	—	6 (2–7)
Living donor, *n* (%)	—	—	—	2 (67%)	—	—	—	3 (100%)
Immune treatment, *n* (%)								
Azathioprine	—	—	—	1 (33%)	—	—	—	0
Mycophenolate mofetil	—	—	—	1 (33%)	—	—	—	2 (66%)
Calcineurin inhibitors	—	—	—	3 (100%)	—	—	—	3 (100%)
Serology pre-vaccination								
S1 IgG, BAU/mL (range)	68 (14–743)	37 (12–313)	187 (14–768)	15 (13–80)	0.2 (0.1–1.2)	0.4 (0.1–1.2)	0.9 (0.1–2.7)	0.6 (0.1–1.7)
N IgG, AU/mL (range)	50 (3–242)	30 (12–108)	206 (0–875)	3 (1.0–22)	2.0 (0.1–2.7)	2.8 (1.6–22)	2.9 (1.1–5.4)	1.8 (0–3.3)

^a^The body-mass index (BMI) is the weight in kilograms divided by the square of height in meters. *CKD* chronic kidney disease, *KTR* kidney transplant recipient, *eGFR* estimated glomerular filtration rate, *S1* SARS-CoV-2 spike head domain, *N* nucleocapsid, *BAU* Binding Antibody Units.

### SARS-CoV-2-specific (neutralizing) antibodies are higher in individuals with hybrid immunity

**Fig. 1:**
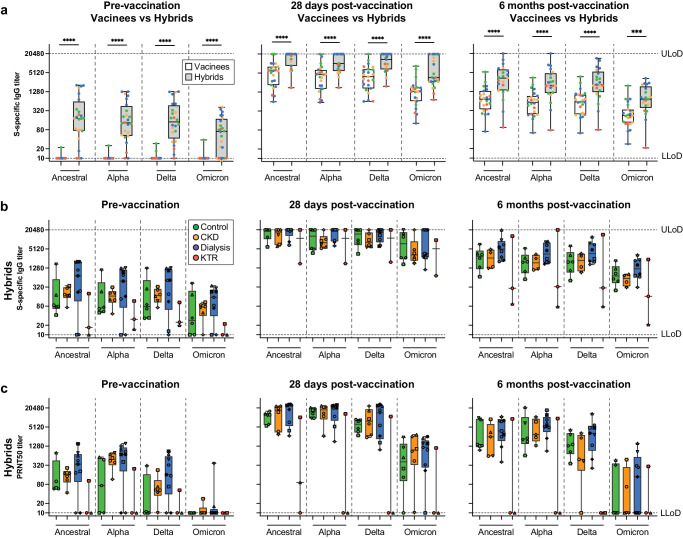
Hybrid immunity induces higher serum SARS-CoV-2 binding and neutralizing antibody titers then vaccination alone. **a** Longitudinal serum spike (S)-specific IgG titers of SARS-CoV-2 variants in vaccinees without (white box), and with prior COVID-19 (hybrids, grey box) sampled before, and 28 days and 6 months after vaccination. Groups are color-coded similar to panels **b** and **c**. **b** S-specific IgG titers to SARS-CoV-2 variants in hybrid immune controls (green), chronic kidney disease patients (CKD, yellow), patients on dialysis (blue) and kidney transplant recipients (KTR, red). The 50% effective concentration serum S-specific IgG titers were determined by ELISA. **c**) The serum antibody titers of a 50% plaque reduction neutralization test (PRNT50) using clinical virus isolates of each variant. Symbols, central line, box and whiskers represent individual patients, the mean, interquartile range and minimum or maximum values, respectively. Horizontal dotted lines indicate the upper (ULoD) and lower limits of detection (LLoD). Significant differences calculated by Mann–Whitney-U test are indicated (***p < 0.005 and ****p < 0.001).

The kinetics of the antibody response towards the ancestral, Alpha, Delta and Omicron BA.1 variant showed similar trends as previously reported^25^, with consistently higher titers in hybrids compared to vaccinees, and with gradually lower titers against antigenically more distinct variants (Fig. 1a). Serum titers of IgG binding antibodies to the control antigens HA and TT remained stable over time for all study participants (Supplementary Fig. 2). In vaccinees, S-specific serum IgG titers showed similar trends as reported previously (Supplementary Fig. 3)^3–5,26^. In hybrids, patients on dialysis tended to have highest S-specific IgG for all variants (Fig. 1b). Overall, the levels of neutralizing antibodies followed similar trends as S-specific IgG (Fig. 1c). Of note, two out of three KTRs did not develop detectable neutralizing antibodies, while they did have low levels of S-specific IgG titers (Fig. 1b, c).

### SARS-CoV-2 S-specific B-cells cluster in IgG-high regions

To investigate the frequency and phenotypic characteristics of B-cells in hybrid immune kidney disease patients and controls, we determined the expression of markers for B-cell differentiation (CD27, CD38, CD24, CD20, CD138), BCR isotype (IgD, IgM, IgA, IgG), lymph node homing (CD62L) and S-specificity on live CD19+ B-cells in PBMC using flow cytometry.

**Fig. 2:**
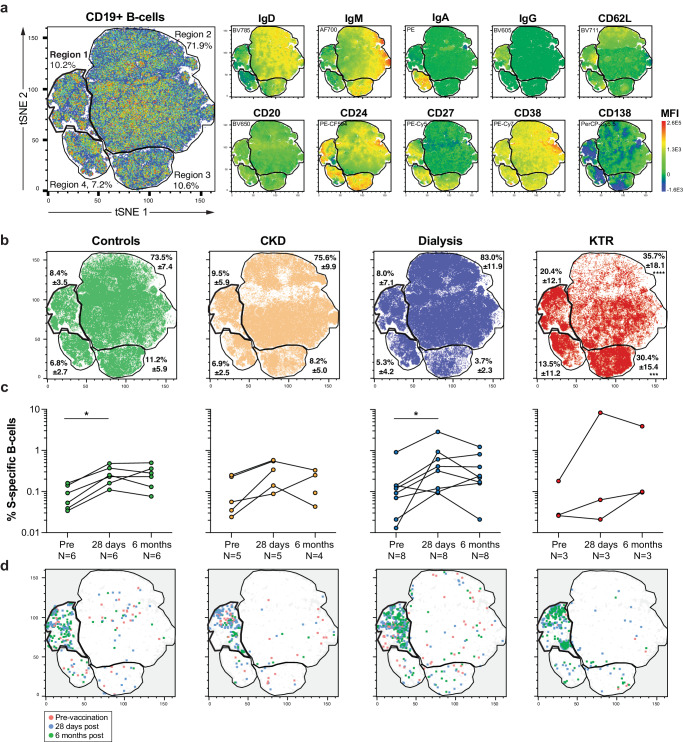
Kinetics and phenotypic analysis of S-specific B-cells in kidney disease patients and controls. **a** A pseudo-colored B-cell density plot (from red to blue, highest to the lowest density of events) of multicolor flow cytometry data after dimensionality reduction by t-distributed stochastic neighbor embedding (t-SNE) from concatenated, normalized, live, single CD19+ events from all individuals and timepoints (left panel) based on the expression of the 10 indicated markers as determined by the mean fluorescent intensity (MFI, right panels). Four dominant regions (Region 1-4) are outlined. **b** Mean and standard deviation of the frequency of events over the 4 regions per study group. Significant differences determined using two-way ANOVA and Tukey’s multiple comparison test are indicated (***p < 0.005 and ****p < 0.0001). **c** Longitudinal frequen**c**ies of live single S-specific CD19+ B-cells identified by classical gating in controls, CKD, dialysis and KTR pre-, 28 days and 6 months post-vaccination. Significant differences determined by Wilcoxon test for paired data are indicated (*p < 0.05). **d**) S-specific B-cells were overlayed on the t-SNE plot and depicted separately per study group and color-coded timepoint (red, blue and green).

B-cells from hybrid controls and kidney disease patients at all time points segregated into four dominant regions based on BCR isotype (Fig. 2a). Region 1 had the highest expression of IgG, as determined by the mean fluorescence intensity (MFI). Region 2 had the highest expression of IgD and IgM, region 3 of IgM, and region 4 of IgA. Overall, regions 1, 3, and 4 showed the highest expression of CD27, indicating that class-switched B-cells with canonical memory marker expression were dominant in these 3 regions. Expression of other markers had a more diffuse pattern (Fig. 2a). Each region contained CD19+ B-cells from all patient groups (Fig. 2b), but these cells segregated differently between groups. KTR had a noticeable lower density of events in the CD27-low region 2 and more in IgM-high region 3 compared to other groups. This suggested KTR have lower frequencies of naïve and more IgM B-cell phenotypes. Other minor differences in the distribution of events within clusters were mainly explained by donor variation and not by group-specific phenotypic differences (data not shown).

The percentage of S-specific live CD19+ B-cells detected 28 days after vaccination was consistently higher than prior to vaccination in all participants, except for one KTR. This increase was statistically significant for controls and patients on dialysis (Fig. 2c). In contrast to a decrease of antibody levels at 6 months post-vaccination, the percentage of S-specific live CD19+ cells did not differ from the percentage at 28 days post-vaccination. S-specific B-cells frequencies did not differ between groups (Supplementary Fig. 4).

Because of the low frequency of circulating S-specific B-cells, phenotypic analysis was performed in the context of neighboring cells with a similar phenotype but different specificity (Fig. 2b). We did not find specific characteristics in the distribution of S-specific B-cells across time points in any of the kidney disease groups nor controls. Instead, S-specific B-cells were predominantly found in the IgG-high region 1 in all groups across time points (Fig. 2d).

### SARS-CoV-2-specific B-cells have variable phenotypes within and between patient groups

**Fig. 3:**
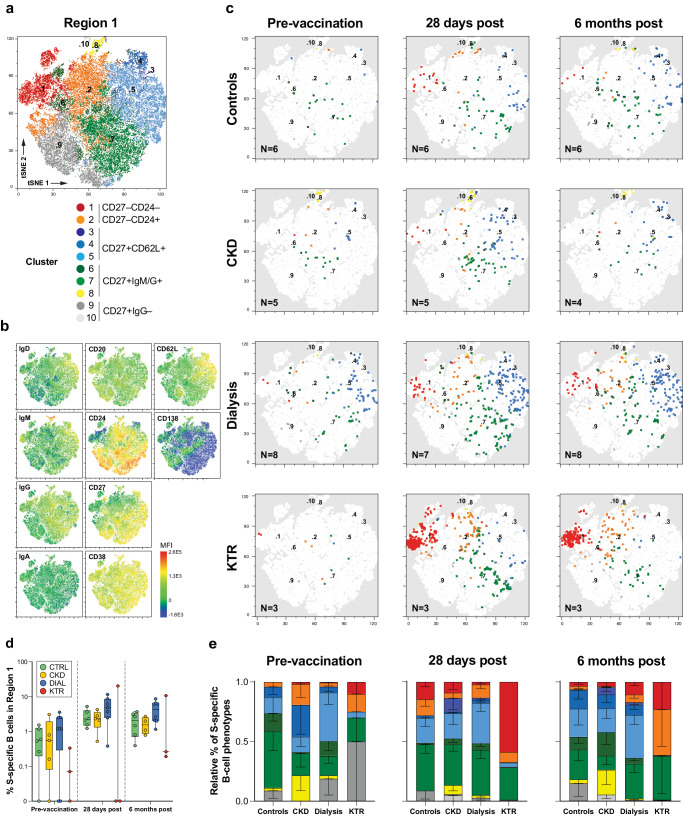
S-specific B-cells in hybrid kidney disease patients and controls display subtle phenotypical variations. **a** t-SNE-based dimensional reduction plots of concatenated B-cells in region 1 (Fig. 2a). Clusters identified by unsupervised cluster analysis using FlowSOM are numbered in the center of each cluster and color coded according to their phenotypic resemblance; CD27+ B-cells with low Ig expression (cluster 9,10; greys), class-switched CD27+ memory populations (cluster 6-8; yellow/greens), CD62L+ lymph node homing (cluster 3–5; blues) and CD27– extrafolicular memory populations (cluster 1,2; orange and red). **b** S-specific B-cells of hybrid controls, CKD and dialysis patients, and KTR overlayed on the t-SNE plot, color coded per cluster. **c**) Distribution of isotype, memory and lymph node homing marker expression shown as the mean fluorescent intensity (MFI) on the t-SNE map. **d** Frequency of S-specific B-cells within region 1. The central line, box, and whiskers represent the mean, interquartile range, and minimum or maximum values, respectively. **e** Relative mean frequencies (boxes) and standard error of mean (whiskers) of S-specific B-cell phenotypes in region 1, color-coded similar to panel **a**. Samples without S-specific B-cells in region 1 were omitted from this analysis.

To further investigate phenotypic differences in S-specific B-cells between patient groups and controls, we zoomed in on the IgG-high region 1. A t-SNE plot of all events in region 1 was generated to improve resolution. Using FlowSOM, we identified 10 clusters of B-cells with differential surface marker expression in region 1 and overlayed them on the t-SNE plot (Fig. 3a, b). Subsequently, S-specific B-cells were traced back in each FlowSOM cluster and classified per disease group and time point (Fig. 3a, c). We observed similar trends in kinetics for S-specific B-cells in region 1 (Fig. 3d) compared to all S-specific B-cells (Fig. 2c) and to serology (Fig. 1b); responses in patients on dialysis tended to be the highest.

Within region 1, we consistently observed S-specific B-cells with classical CD27+IgM+ or CD27+IgG+ memory phenotypes in all patient groups. Although the relative proportion of these phenotypes was slightly lower in kidney disease groups compared to controls at baseline, this difference between groups became smaller over time (Fig. 3a, c and e; cluster 6–8, yellow and green colors, respectively). Additionally, S-specific B-cells with CD27+CD62L+ lymph node homing phenotypes were observed. However, these were rarely detected in KTR (Fig. 3a, c and e; cluster 3-5, blue colors). Two different class-switched S-specific B-cell phenotypes lacking the canonical memory marker CD27 were additionally detected in approximately half of the participants. These consisted of CD27–CD24+IgG+ S-specific B-cells and CD27–CD24– B-cells (Fig. 3a, c and e; cluster 1–2, orange and red color). The latter are typically referred to as class-switched double negative B-cells^27^. These phenotypes were a minority at all time points, except for one KTR, who developed the highest number of S-specific B-cells which were predominantly of a double negative phenotype.

The relative frequencies of the CD27+IgG/M+ , CD27+ CD62L+ , CD27–CD24– and CD27–CD24+ phenotypes identified in the t-SNE, which was generated using down-sampling to normalize B-cell counts for all donors, were analyzed on the full data set using classical gating. Here, we found similar distributions of the defined phenotypes with slightly decreased standard deviations due to increased B-cell sample size, confirming our findings based on dimensional reduction (data not shown).

### Phenotype of SARS-CoV-2-specific T-cells remains similar over time

**Fig. 4:**
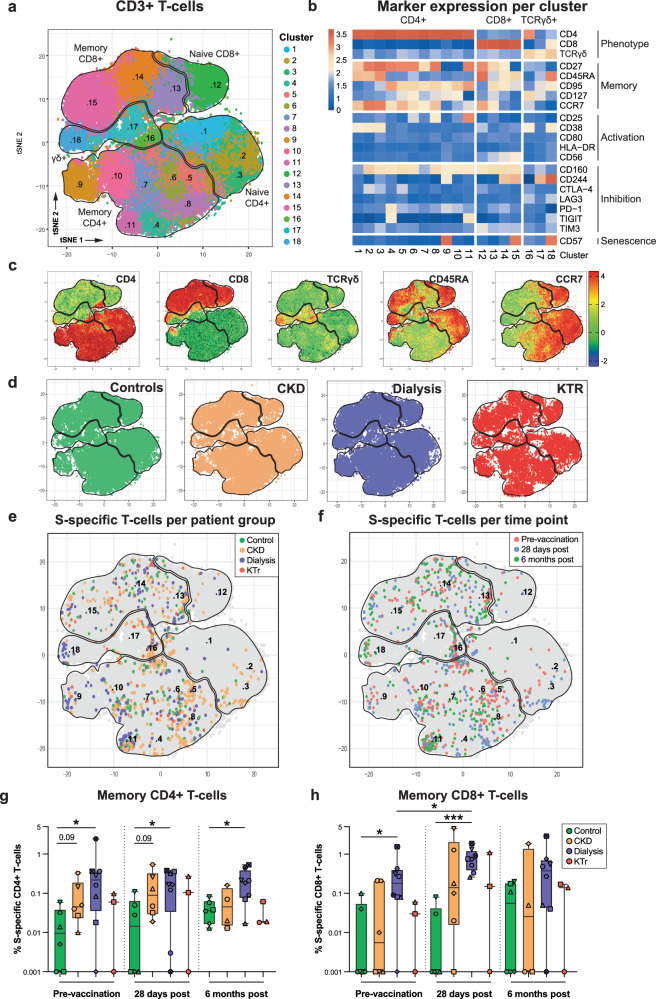
S-specific T-cells are phenotypically diverse but similar between groups and over time. **a** t-SNE-based dimensional reduction plot of concatenated T-cells, clustered based on the relative expression of phenotypic, memory, activation, inhibition and senescence markers, color coded per cluster. **b** Heatmap of relative marker expression per cluster. **c**) Populations of naive and memory CD4+ and CD8+ T-cells, and γδT-cells are outlined based on differential expression of CD4, CD8, TCRγδ, CD45RA and CCR7 shown by the mean fluorescent intensity (MFI). **d** Distribution of concatenated T-cells per patient group. **e** S-specific CD4+ , CD8+ T-cells and γδT-cells are overlayed and color coded per patient group or **f** per time point. **g** The frequency of memory CD4+ and **h**) CD8+ T-cells. The symbols, central line, box and whiskers represent individual patients, mean, interquartile range and minumum and maximum values, respectively. Significant differences calculated by Mann-Whitney-U test are indicated (*p < 0.05 and ***p < 0.005).

Next, we performed t-SNE dimensionality reduction on T-cells gated from PBMC based on the expression of phenotypic markers (CD4, CD8, γδTCR), memory markers (CD27, CD45RA, CD95, CD127, CCR7), activation markers (CD25, CD38, CD80, HLA-DR), inhibitory markers (CD160, CD244, CTLA-4, LAG3, PD-1, TIGIT, TIM3), and a marker for senescence (CD57). This effectively segregated the T-cells into 3 main regions on the t-SNE, specifically, CD4+ , CD8+ , and γδTCR+ T-cells (Fig. 4a, b and Supplementary Fig. 5). Within the CD4+ and CD8+ regions, we visually identified subregions of naive (CD45RA+ CCR7+ ) and memory (CD45RA–CCR7+ , CD45RA–CCR7– or CD45RA+ CCR7–) T-cells (Fig. 4a, c). Using unsupervised clustering analysis, eighteen clusters were identified that consisted of eleven CD4+ phenotypes (cluster 1-11), four CD8+ phenotypes (cluster 12-15), and three γδTCR+ phenotypes (cluster 16-18, Fig. 4a, b). T-cells did not differentially cluster over the identified phenotypes for either the different patient groups or per time point (Fig. 4d).

To further phenotype the S-specific T-cells, we traced back CD4+CD137+OX40+ and CD8+CD137+CD69+ T-cells on the t-SNE plot for each study group and time point (Fig. 4e, f). SARS-CoV-2 S-specific T-cells did not differentially cluster over the identified phenotypes, neither per patient group, nor per time point. However, S-specific T-cells were almost exclusively identified in the memory CD4+ T-cells (cluster 4-11), memory CD8+ T-cells (cluster 13–15), and γδTCR+ T-cells (cluster 16-18) (Fig. 4e, f). Next, we assessed the kinetics of S-specific memory T-cells across groups by quantifying the frequency of CD4+OX40+CD137+ and CD8+CD69+CD137+ T-cells in longitudinal samples. Surprisingly, controls had relatively low frequencies of S-specific memory CD4+ and CD8+ T-cells pre-vaccination and post-vaccination compared to kidney disease groups, with significantly higher frequencies in patients on dialysis. S-specific T-cells were consistently detected in two out of three KTR (Fig. 4g, h). In contrast to S-specific B-cells, mRNA-1273 vaccination did not boost the S-specific memory CD4+ T-cell response in any group. However, S-specific memory CD8+ T-cells were boosted in patients on dialysis.

To further explore phenotypic differences over time and between study groups, we conducted unsupervised dimensionality reduction on a concatenated file containing either all CD4+ or CD8+ SARS-CoV-2 S-specific T-cells. We identified 12 clusters for CD4+CD137+OX40+ or CD8+CD137+CD69+ T-cells based on the differential expression of phenotypic, memory, activation, inhibition and senescence markers (Supplementary Fig. 6a, b, e and f). No significant differences were observed in the distribution of S-specific T-cells between study groups or over time (Supplementary Fig. 6c, d, g and h). Overall, our findings suggest that the phenotype of SARS-CoV-2-specific T-cells is highly diverse, with no consistent patterns observed between patient groups and controls, or between time points. Of note, no significant enrichment of T-cells expressing markers correlated with exhaustion (e.g., clusters 4, 9, 12, 14-15, and 17-18 based on high PD-1, TIGIT, TIM-3 and CD244 expression) or senescence (CD57-high clusters 9, 15 and 18 in Fig. 4b and cluster 2 in Supplementary Fig. 6b) was observed for the different patient groups (Fig. 4e, Supplementary Fig. 6c).

### Humoral and cellular immune responses correlate within their respective compartments

**Fig. 5:**
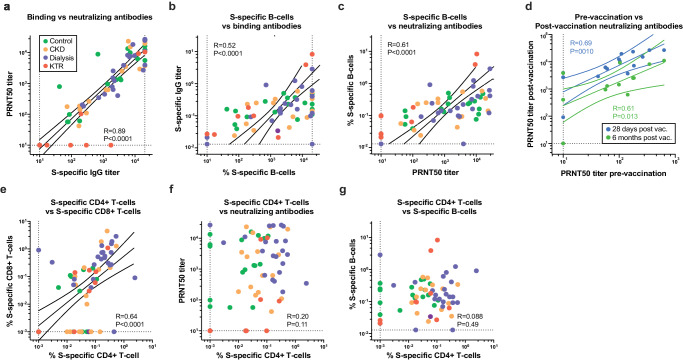
B- and T-cell responses correlated within their own compartment, but do not correlate with each other. **a–g** Linear regression was performed on 10log-transformed data from all available patients and timepoints. Spearman rank correlation was used to calculate R and significance. For binding IgG titers and PRNT50, data of the ancestral lineage is shown. Only for significant correlations, regression, and 95% confidence intervals are plotted. Horizontal and vertical dotted lines depict the LLoD and ULoD.

To investigate whether the measured SARS-CoV-2-specific B-cell and T-cell responses correlated, we performed linear regressions on log-transformed antibody titers, and frequencies of S-specific B-cells, CD4+ T-cells and CD8+ T-cells (Fig. 5, Supplementary Fig. 7). S-specific IgG and neutralizing titers were significantly correlated (Fig. 5a). When correlating ancestral binding or neutralizing antibodies with variant-specific neutralization, the lowest regression was seen for the Omicron BA.1 variant (Supplementary Fig. 7a and b).

The frequency of S-specific B-cells correlated with IgG binding and neutralizing antibodies (Fig. 5b, c). Moreover, levels of neutralizing antibodies prior to vaccination correlated with neutralizing titers after vaccination, confirming that higher neutralization titers induced by SARS-CoV-2 infection set a pre-disposition to develop stronger neutralizing responses after mRNA-1273 vaccination (Fig. 5d). To assess the contribution of the different S-specific B-cell populations to the development of neutralizing titers, we correlated individual and grouped S-specific B-cell FlowSOM population frequencies of region 1 (defined in Fig. 3a) with PRNT50 titers. Significant correlations were detected for CD27–CD24+/CD27–CD24– (clusters 1 and 2), CD27+CD62L+ (clusters 3-5), and CD27+IgM/G+ B-cells (cluster 6-8), as well as for the individual clusters 5 and 7 (Supplementary Fig. 7C).

When comparing SARS-CoV-2-specific CD4+ and CD8+ T-cell responses, a significant correlation was observed. However, a fraction of the participants had SARS-CoV-2 specific CD4+ T-cell responses in the absence of SARS-CoV-2 specific CD8+ T-cell responses (Fig. 5e). No significant correlations between SARS-CoV-2-specific B- and T-cell responses, or neutralizing antibodies and SARS-CoV-2-specific T-cell responses, were observed (Fig. 5f, g).

## Discussion

Here, we investigated longitudinal SARS-CoV-2-specific antibody, B-cell, and T-cell responses in a small group of kidney disease patients with hybrid immunity acquired by asymptomatic or mild infection followed by vaccination, and compared in-depth immunological responses of these kidney patients to control participants with hybrid immunity. To our knowledge, our study is the first to describe antigen-specific T-cell and B-cell phenotypes and the interplay between immune compartments in hybrid immune kidney patients. In conclusion, we show that, first, hybrids have more potent S-specific immune responses than vaccinees. Second, hybrid dialysis patients tend to have stronger SARS-CoV-2-specific immune responses compared to hybrid controls. Third, the virus-specific B- and T-cell response are phenotypically diverse across patient groups. Fourth, there are subtle phenotypic differences within and between study groups in SARS-CoV-2-specific B-cells. Fifth, multiple antigen exposures did not lead to a higher level of S-specific T-cells with an exhausted phenotype in kidney patients.

Hybrid immune groups had significantly stronger SARS-CoV-2-specific antibody responses compared to vaccinees. This extended towards reactivity with the studied SARS-CoV-2 variants, supporting that hybrid immunity provides a more potent response compared to vaccination-induced immunity alone. Although genome sequencing data was not available for hybrids, these were all infected prior to the introduction of vaccines, likely the ancestral or alpha SARS-CoV-2 variant, which are antigenically similar^10^.

The magnitude of immune responses was strikingly similar between hybrid CKD G4/5, dialysis patients, and hybrid controls. However, this study was underpowered to determine if this was also the case for KTR. On average, hybrid patients on dialysis had higher T-cell frequencies and tended to have higher S- and N-specific antibody titers and B-cell frequencies compared to hybrid controls at all time points. The increased response in patients on dialysis prior to vaccination could either be explained by a more recent SARS-CoV-2 infection, or by an increased response to infection due to disease- or medication-specific immune alterations^28,29^. In contrast to mRNA-1273 vaccination, which does not self-amplify, SARS-CoV-2 infection might lead to increased or prolonged viral replication and increased antigen expression in immune-compromised individuals compared to controls^30,31^. This may explain similar or increased adaptive immune response in specific immunocompromised groups compared to controls, who more effectively control viral replication in the absence of differences in clinical disease presentation^32,33^.

Although the phenotypes of S-specific T-cells were consistent across time and between hybrid kidney disease patient groups and controls, we detected subtle phenotypic differences in the distribution of the overall B-cell population and of S-specific B-cells. KTR showed decreased frequencies of the IgD+CD27– B-cells and increased frequencies of IgM+ , IgA+ and IgG+ class switched B-cells, which is likely associated with the use of calcineurin inhibitors^34^. For S-specific B-cells, the observed differences in relative frequencies of class-switched memory and lymph node homing B-cell phenotypes between groups observed at baseline indicate that these individuals responded differently to SARS-CoV-2 infection. However, as these differences normalized over time after vaccination for CKD G4/5 and dialysis patients, this indicates that repeated mRNA-1273 immunization can mitigate these differences. Responses in KTRs were markedly different from the other groups and varied greatly within this group. Potentially this reflects the variation in time since transplantation and use of immunosuppressive drugs. A minor proportion of class-switched double-negative S-specific B-cells were detected before vaccination in controls, CKD G4/5, and dialysis patients prior to vaccination, yet all groups developed this phenotype after vaccination that persisted over time. B-cells with a double-negative phenotype have been associated with extrafollicular responses and with autoimmunity^27^. In contrast to kidney disease patients, the control group displayed a low relative frequency of CD27– and CD62L+ S-specific B-cell populations and high CD27+ S-specific B-cell fraction, indicating a more extensive germinal-center training of these S-specific B-cells^35^. As opposed to germinal center-derived B-cells, extrafollicular B-cells undergo a faster development and are more prone to differentiate into antibody-secreting cells^36^. The level of somatic hypermutation in the BCR and affinity maturation of double-negative B-cells as compared to typical memory CD27+ B-cells is limited^37^. However, in critically ill COVID-19 patients, the development of neutralizing antibodies was correlated with the presence of double-negative B-cells^27^. In line with these findings, the only KTR that developed strong antibody binding and neutralizing titers also displayed a prominent level of S-specific B-cells with a double-negative phenotype. In addition, this KTR also developed S-specific CD4+ and CD8+ T-cells. This intra-group variation could be explained by the 19-year period after transplantation, which was accompanied with decreased use of immunosuppressive drugs for this individual, compared to less than a 10-year period for the two other KTRs^38,39^.

Prior to vaccination, most hybrid immune individuals had SARS-CoV-2-specific CD4+ and CD8+ T-cell responses. All the hybrid kidney disease patients, including KTRs, and control individuals established a sustained S-specific CD4+ T-cell response. Interestingly, circulating S-specific CD4+ T cell frequencies appeared to be decoupled from the humoral and S-specific B-cell response. Unlike S-specific B-cells, CD4+ T-cell frequencies were not boosted by vaccination, which is consistent with previous studies^40–42^. S-specific CD8+ T-cell frequencies did increase after vaccination. However, an S-specific CD8+ T-cell response was not detected in all individuals. This data is in line with previous research, in which healthy hybrid individuals induced S-specific CD4+ but low to no detectable CD8+ T-cells after one or two doses mRNA vaccination^43^. In addition, all KTRs develop S-specific CD4+ T-cells overtime, but two out of three did not develop a high antibody and B-cell response. These individuals could be protected from severe COVID-19 by a strong virus-specific T-cell response in the absence of a B-cell response, considering that the included hybrid KTR only experienced mild to asymptomatic SARS-CoV-2 infection^44–47^.

The phenotype of CD4+ and CD8 + SARS-CoV-2 specific T-cells proved to be highly diverse based on differential expression of markers for memory, activation, inhibition and senescence. Nevertheless, similar frequencies of S-specific T-cells were identified in these clusters within study groups. Consistency in the phenotype of the S-specific T-cell response could indicate that T-cells were effectively induced by the priming infection for most participants, and that vaccination did not alter pre-existing phenotypes. Several studies described that T-cells from kidney disease patients, especially patients on dialysis, tend to have an exhausted phenotype due to constant exposure to uremic toxins^48–50^. However, although specific clusters of exhausted T-cells were identified, these did not differ between study groups and over time.

A major limitation of our study is the group size. This constrains generalizability of our findings and extrapolation of our observations to individuals suffering from kidney disease, making this an observational study. All hybrid immune individuals included in this study had mild or asymptomatic COVID-19 due to infection with the ancestral or alpha variant of SARS-CoV-2 prior to vaccination. As the mortality and disease severity in kidney disease patients is generally more severe, our cohort could suffer from a survivorship or severity selection bias^51^. Consequently, our findings cannot be extrapolated to kidney disease patients who suffered from severe COVID-19 prior to vaccination nor to patients that experience breakthrough infection after vaccination, which is often related to more recent and antigenically distinct variants. Another potential limitation to consider is the exclusion of patients undergoing immunosuppressive therapy from the CKD G4/5 and dialysis cohorts. This exclusion, while aimed at specifically assessing impaired kidney function and replacement treatment, may influence the observed seroconversion rates in these groups. Therefore, caution is warranted in extrapolating our findings to individuals on immunosuppressive therapy, highlighting the need for future research in this patient subset.

In conclusion, although mRNA-1273 is less immunogenic in kidney disease patients compared to controls, in kidney disease patients with prior asymptomatic or mild COVID-19, mRNA-1273 has the potential to boost strong humoral and cellular immune responses that are phenotypically similar to those in controls. However, the heterogeneity in the humoral immune responses within and between hybrid immune kidney patient groups warrants further studies on virus-specific immunity in larger kidney disease cohorts. Potentially, this will aid to unravel markers of long-term protection that can be used for personalized vaccination regimens. Given the circulation of evolving SARS-CoV-2 immune escape variants and the introduction of updated vaccines that continuously increase the variation in immunological background, a key challenge will be to identify well-defined study cohorts of kidney disease patients and controls to delineate infection and vaccination effects.

## Methods

### Study participants

Control subjects (without kidney disease, estimated glomerular filtration rate (eGFR) > 45 mL/min/1.73 m^2^), CKD G4/G5 (eGFR <30 mL/min/1.73 m^2^) patients, patients on dialysis, including hemodialysis and peritoneal dialysis, and KTR were included from the RECOVAC-IR study with initial enrollment from February to May, 2021^3^. Hybrid immune individuals (hybrids) were defined as COVID-19 convalescent prior to vaccination, having a SARS-CoV-2 S1-specific antibody level of ≥10 binding antibody units per mL (BAU/mL) at baseline^3^. All participants either experienced asymptomatic or mild COVID-19 symptoms. For serological analysis (but not the cellular analysis), participants with no prior reported SARS-CoV-2 infection were included as comparator, who were SARS-CoV-2 S1 seronegative at baseline (vaccinees). Vaccinees were age- and sex-matched to hybrids. We excluded participants who experienced a breakthrough SARS-CoV-2 infection after vaccination, as determined through questionnaires. All participants received two mRNA-1273 vaccinations (Moderna® Biotech Spain, S.L.) with an interval of 28 days. To assess SARS-CoV-2-specific immune responses, cryopreserved blood samples that were collected at baseline, and 28 days and 6 months after second vaccination were analyzed.

### SARS-CoV-2 variant and control antigens ELISA

SARS-CoV-2 infection prior to vaccination was detected using validated fluorescent bead-based multiplex-immunoassay SARS-CoV-2 Spike S1-specific and nucleoprotein (N) IgG antibodies (Wuhan isolate) as previously described^3,52^. For S1, concentrations were normalized to binding antibody units (BAU) per ml by interpolating values from NIBSC/WHO COVID-19 reference serum 20/136, using a 5-parameter logistic fit. Arbitrary units (AU/mL) were used for N-specific antibodies. For subsequent serological assays, an in-house ELISA was used for the quantification of IgG levels specific for SARS-CoV-2 S trimers and control antigens^53^. In brief, SARS-CoV-2 S trimers of the ancestral (D614G), Alpha, Delta and Omicron BA.1 variant, ancestral SARS-CoV-2 nucleocapsid (N, all Sino Biological), hemagglutinin from pandemic 2009 H1 influenza virus (HA, Sino Biological), and tetanus toxoid (TT, Calbiochem) were coated on Corning Costar 96-Well EIA/RIA plates in PBS overnight at 4 °C at specified concentrations (Supplementary Table 1). ELISA plates were washed with PBS supplemented with 0.05% Tween 20 (washing buffer) and blocked with washing buffer supplemented with 0.1% BSA (blocking buffer) for 1 hour at 37 °C. A dilution series of serum samples was prepared in a blocking buffer. Serially diluted sera were added to the coated and blocked ELISA plates and incubated for 1 hour at 37 °C. Next, plates were washed three times with washing buffer and incubated with HRP-goat anti-human IgG diluted in blocking buffer for 1 hour at 37 °C. After incubation, plates were washed three times with washing buffer and incubated with a 3,3′,5,5′-tetramethylbenzidine solution (Invitrogen, Thermo Fisher Scientific) for 10 minutes. The peroxidase reaction was stopped with 0.5 N sulfuric acid. ELISA plates were scanned for OD at 450 nm with 620 nm reference in a Tecan Infinite F200 reader. 50% of the effective concentration (EC50) titer, as referred to as S-specific IgG titer, was calculated using Graphpad Prism v.9.

### Virus neutralization assay

Serum samples were tested for neutralization capacity against SARS-CoV-2 variants as previously described^25,54,55^. Sequence verified ancestral (D614G), Alpha, Delta, and Omicron BA.1 variants were isolated and cultured from diagnostic specimen and subsequently used in neutralization assays (GenBank accession numbers OM304632, MW947280, OM287123, OM287553, respectively). Briefly, heat-inactivated sera were diluted in OptiMEM medium (Gibco) supplemented with 100 IU penicillin and 100 µg streptomycin (Westburg) starting at a dilution of 1:10, followed by two-fold serial dilutions. 60 µl of diluted serum was transferred to a 96-well V-bottom plate in duplicate. Subsequently, 60 µl of virus suspension was added to each well and incubated for 1 hour at 37 °C. After 1 hour, mixtures were transferred to Calu-3 cells seeded in a 96-well F-bottom plate and incubated for 8 h at 37 °C. Cells were fixed with 10% formaldehyde and stained using a polyclonal rabbit anti-SARS-CoV-2 N antibody (Sino Biological) and a secondary peroxidase-labelled goat anti-rabbit IgG (Dako). Infected cells were visualized using 3,3’,5,5’-tetramethylbenzidine substrate (TrueBlue; Kirkehaard & Perry Laboratories). The number of infected cells per well (plaques) was counted using an ImmunoSpot Image Analyzer (CTL Europe GmbH). The dilution that yielded a 50% reduction of plaques compared with the infection control (PRNT50) was determined by the proportionate distance between two dilutions from which an endpoint titer was calculated.

### Flow cytometry—B-cells

For the identification of S-specific B-cells by flow cytometry, trimerized Wuhan-1 S (Miltenyi) containing a biotin tag was labeled with two fluorescent tags; either Streptavidin-AlexaFluor647 or Streptavidin-BB515 for 1 hour at 4 °C, as previously described^56^. After incubation, additional biotin was added to the mixes and incubated for 15 minutes at 4 °C to quench free fluorochrome-labeled Streptavidin. For phenotypic analysis of B-cell populations, an antibody mix with fluorescently conjugated monoclonal antibodies targeting the following surface molecules was prepared: CD3-BV510 (clone UCHT1, BD Biosciences), CD14-BV510 (clone M5E2, Biolegend), CD16-BV510 (clone 3G8, BioLegend), CD19-APC/Fire750 (clone HIB19, BioLegend), CD20-BV650 (clone L27, BD Biosciences), CD24-PE/Dazzle594 (clone ML5, BioLengend), CD27-PE/Cyanine5 (clone M-T271, BioLegend), CD38-PE/Cyanine7 (clone HIT2, BioLegend), CD62L-BV711 (clone SK11, BD Biosciences), CD138-PerCP-Cy5.5 (clone MI15, BD Biosciences), IgD-BV785 (clone IA6-2, MACS Miltenyi), IgM-AlexaFluor700 (clone MHM-88, BioLegend), IgG-BV605 (clone G18-145, BD Biosciences). Further details on used antibodies are provided in Supplementary Table 1. The viability dye 405 nm LIVE/DEAD Fixable Aqua Dead Cell Stain (Invitrogen) was included in the antibody mix. Peripheral blood mononuclear cells (PBMCs) were thawed in Gibco Roswell Park Memorial Institute 1640 medium (RPMI, Gibco) supplemented with 10% human serum (Sanquin, Rotterdam; R10H), penicillin (100 IU/ml; Lonza, Belgium), streptomycin (100 μg/ml; Lonza, Belgium), and 2 mM L-glutamine (Lonza, Belgium; R10H medium), and treated with Benzonase (50 IU/ml; Merck) at 37 °C for 30 min. PBMC were resuspended in PBS supplemented with 0.05% BSA (Sigma Aldrich) and 0.05 mM EDTA (Invitrogen) containing 5 ml of Brilliant Stain Buffer Plus (BD Biosciences). Next, cells were incubated with the antibody mix for B-cell phenotyping, S-AlexaFluor647 and S-BB515 for 30 minutes at 4 °C. After incubation, free antibodies and antigen baits were washed with PBS supplemented with 0.05% BSA (Sigma Aldrich) and 0.05 mM EDTA (Invitrogen) buffer, and cells were fixed overnight at 4 °C in 2% paraformaldehyde (PFA) until cytometric analysis. Acquisition of B-cell populations was performed using a BD FACS Fortessa (BD Biosciences).

### Phenotypic analysis of SARS-CoV-2 specific B-cells

Multicolor flow cytometry data files were analyzed on FlowJo software v.10. First, live/singlets, CD3–CD14–CD16–CD19+ events were gated by classical gating (identified as B-cells) (Supplementary Fig. 1a). Next, a concatenated file with a down-sampled normalized number of B-cells per donors and time points was generated to proceed with the phenotypic analysis of B-cells. Dimensionality reduction was performed by generating t-distributed stochastic neighbor embedding (t-SNE) plots based on all fluorescent parameters related to phenotype (CD20, CD24, CD27, CD38, CD138, IgA, IgD, IgG, IgM), excluding S-specific antigen-binding. An exact K-nearest neighbors (KNN) and Barnes-Hut gradient algorithm with a perplexity of 30 were used to generate t-SNE plots. S-specific B-cells were identified by classical gating on Spike-positive AlexaFluor647+BB515+ events (Supplementary Fig. 1a) and traced back on the t-SNE plots to identify their phenotype. Maps of S-specific B-cells on t-SNE plots were analyzed separately per donor group and time point to study intrinsic group characteristics and kinetics of the S-specific B-cell response. Subsequently, the region with the highest density of S-specific events was selected by classical gating and distributed on a new t-SNE plot for better segregation of events. Within these t-SNE plots, populations of closely related B-cells based on their expression of surface markers were identified using FlowSOM algorithm (v.2.5.2).

### Flow cytometry—T-cells

For T-cell analysis, 4 × 10^6^ thawed PBMCs were transferred to U-bottom plates in 100 µl R10H to rest at 37 °C. After 6 h, PBMCs were stimulated for 20 h at 37 °C with an overlapping peptide pool (15 oligomers with 11 amino acids overlap) spanning the entire ancestral S protein (315 peptides, PepMix; JPT) at a concentration of 1 µg/ml for the detection of SARS-CoV-2-specific T-cells. As a negative control, PBMCs were stimulated with an equimolar concentration of DMSO. After stimulation, PBMCs were washed twice with FacsWash (FW) consisting of HBSS (Life Technologies), 0.02% NaN_3_ (Merck Life Science), and 3% heat-inactivated FBS. For phenotypic T-cell analysis, cells were transferred to true stain monocyte blocker (BioLegend, San Diego, CA, USA) and Brilliant Stain Buffer Plus (BD) before surface marker staining. Subsequently, cells were stained on ice with a panel of 26 fluorescently conjugated monoclonal antibodies in a final volume of 100 µl. Antibodies specific for γδTCR-PerCPeF710 (clone B1.1; ThermoFisher), CD183/CXCR3-BUV563 (clone 1C6/CXCR3; BD) and CD197/CCR7-BV786 (clone 2-L1-A; BD) were individually added with 10–15-minute intervals. Next, antibodies specific for AnnexinV-AF350 (ThermoFisher); CD56-BUV395 (clone B159; BD); CD80-BUV615 (clone L307.4; BD); CD366/TIM3-BUV737 (clone 7D3; BD); CD8-BUV805 (clone SK1; BD); CD152/CTLA4-BV421 (clone BNI3; BioLegend); CD223/LAG3-BV480 (clone T47-530; BD); HLA-DR-BV570 (clone L243; BioLegend); CD134/OX40-BV605 (clone L106; BD); CD69-BV650 (clone FN50; BioLegend); TIGIT-BV711 (clone 741182; BD); CD279/PD-1-BV750 (clone EHI2.2H7; BioLegend); CD95-BB700 (clone DX2; BD) and CD137/41BB-PE (clone 4B4-1; Miltenyi) were sequentially added. Finally, a mix of CD3-Sparkblue550 (clone SK7; BioLegend), CD4-cFluorYG584 (clone SK3; Cytek), CD25-eFluor450 (clone CD25-4E3; life technologies), CD27-VioBright-FITC (clone M-T271; Miltenyi), CD38-APC/Fire810 (clone HIT2; BioLegend), CD45RA-SparkNIR685 (clone HI100; BioLegend), CD57-APC (clone HNK-1; BioLegend), CD127-APC/R700 (clone HIL-7R-M21; BD), CD160-PE/Cy7 (clone BY55; BioLegend) and CD244-PE/Cy5.5 (clone eBioC1.7; ThermoFisher) was added and incubated for 30 min. After staining, cells were washed twice with FW, followed by fixation with 1% PFA overnight at 4 °C before cytometric analysis. All solutions used contained 2.5 mM CaCl_2_ needed for AnnexinV binding. Acquisition of T-cell populations was performed using a 5-laser Aurora spectral flow cytometer (Cytek Biosciences, CA).

### Phenotypic analysis of SARS-CoV-2 specific T-cells

T-cell populations were initially analyzed via classical gating using FlowJo software v10. On average, ~1.000.000 cells were acquired per sample. Cells were gated for CD3+ live/singlets (T-cells) (Supplementary Fig. 1b). Memory CD4+ and CD8+ T-cells were identified and quantified by classical gating. SARS-CoV-2 specific memory CD4+ T-cells were identified by gating CD137+OX40+ events, and SARS-CoV-2 specific memory CD8+ T-cells were identified by gating CD137+CD69+ events (Supplementary Fig. 1c). The DMSO treated sample of the same participant was used to set the cutoff gate for activation markers, and the background measured in the DMSO treated sample was subtracted from the S-stimulated sample. To further phenotype the T-cells, unsupervised cluster analysis was conducted using Self-Organizing Maps (SOM) through the CytoTree (v.1.6.0) and FlowSOM (v.2.5.2) packages in R (v.4.2.1). The data from all JPT stimulated FCS files were concatenated, normalized and logicle transformed. Subsequently, a SOM with 18 clusters was created using the expression matrices containing phenotypic markers (CD4, CD8, γδTCR) memory markers (CD27, CD45RA, CD95, CD127, CCR7), activation markers (CD25, CD38, CD80, HLA-DR), inhibitory markers (CD160, CD244, CTLA-4, LAG3, PD-1, TIGIT, TIM3), and a marker for senescence (CD57). Antigen-specific activation markers (CD69, CD137, and OX40) were excluded. Principal component analysis and dimensionality reduction on the first 50 principal components was performed to generate t-SNE plots. Subsequently, we assigned a naïve or memory CD4+ or CD8+ , or γδ-T-cell phenotype classification to the clusters based on expression profiles. SARS-CoV-2-specific CD4+ and CD8+ T-cells based on the expression of CD137+OX40+ and CD137+CD69+ , respectively, were traced back on the t-SNE plots. Independent expression matrixes were additionally generated containing the classically gated CD4+OX40+CD137+ or CD8+CD69+OX40+ SARS-CoV-2-specific memory T-cells using similar SOM analysis as described above, resulting in 12 clusters each.

### Statistics

Due to the low number of individuals included in this study, the non-parametric Mann-Whitney-U test was used to assess statistical differences in serum IgG titers, PRNT50 titers, S-specific B-cell frequencies, and T-cell frequencies between controls and kidney disease groups, except for KTR. The Wilcoxon test for paired data was used to assess statistical differences in B-cell frequencies between time points. To assess correlations between parameters, log10-transformed data was used for linear regressions and statistical significance and R was assessed by Spearman’s rank correlations test. Differences were considered statistically significant when *p* values were less than 0.05. All statistical analyses were performed with Graphpad Prism 9 (GraphPad Software).

### Study approval

The RECOVAC IR study was approved by the Dutch Central Committee on Research Involving Human Subjects (CCMO, NL76215.042.21) and the local institutional review board of the participating centers (Amsterdam UMC, UMC Groningen, Radboud UMC Nijmegen and Erasmus MC Rotterdam). The study is also registered at clinicaltrials.gov (NCT04741386). All participants provided written informed consent prior to their involvement.

### Reporting summary

Further information on research design is available in the Nature Research Reporting Summary linked to this article.

### Supplementary information

Supplementary Table and Figure 1-7

REPORTING SUMMARY

## Data Availability

Upon a reasonable request, the corresponding authors can supply the data and codes used in this study.
