# Supplementary material for: SARS-CoV-2-specific immune responses converge in kidney disease patients and controls with hybrid immunity
Source: NPJ Vaccines. 2024 May 28;9:93. doi: 10.1038/s41541-024-00886-0 (PMC11133345; doi:10.1038/s41541-024-00886-0)
Supplement: Supplementary file 1 — Supplementary Table and Figure 1-7 [file 41541_2024_886_MOESM1_ESM.pdf]

Supplementary Table: Antigens used in ELISA and antibodies used in phenotypic B- and T-cell analysis.

Antigens for ELISA				
**Antigen**	**Concentration ELISA plates**	**Supplier**	**Cat. Nr.**	
SARS-CoV-2 Ancestral Spike	500ng/ml	Sino Biological	40589-V08H8	
SARS-CoV-2 Alpha Spike	500ng/ml	Sino Biological	40589-V08H12	
SARS-CoV-2 Delta Spike	800ng/ml	Sino Biological	40589-V08H10	
SARS-CoV-2 Omicron Spike	400ng/ml	Sino Biological	40589-V08H26	
SARS-CoV-2 Ancestral Nucleocapsid	800ng/ml	Sino Biological	40588-V08B	
Influenza A pH1N1 (A/Cal/04/09)HA	600ng/ml	Sino Biological	11055-V08H	
Tetanus Toxoid	800ng/ml	Merck	582231-25UG	
**Antigens for flow cytometry – B-cell panel**				
**Antigen**	**Supplier**		**Cat. Nr.**	
SARS-CoV-2 Ancestral Spike	Myltenyi Biotec		130-127-547	
**Antibodies for flow cytometry – B-cell panel**				
**Target**	**Color and dilution**	**Supplier**	**Cat. Nr.**	**Dilution**
Viability	AmCyan (1:100)	Thermo Fisher	L34957	1:100
CD3	BV510 (1:200)	BD	563109	1:200
CD14	BV510 (1:100)	Biolegend	301842	1:100
CD16	BV510 (1:100)	Biolegend	302048	1:100
IgG	BV605 (1:25)	BD	563246	1:25
CD20	BV650 (1:50)	BD	740439	1:50
CD62L	BV711 (1:50)	BD	565040	1:50
IgD	BV785 (1:200)	Biolegend	348242	1:200
Streptavidin	BB515	BD	564453	NA
CD138	PerCP-Cy5.5 (1:50)	BD	341107	1:50
IgA	PE (1:50)	Myltenyi Biotec	130-113-476	1:50
CD24	PE-CF594 (1:50)	Biolegend	311134	1:50
CD27	PE-Cy5 (1:50)	Biolegend	356437	1:50
CD38	PE-Cy7 (1:50)	Biolegend	303515	1:50
Streptavidin	AF647	Biolegend	405226	NA
IgM	AF700 (1:50)	Biolegend	356416	1:50
CD19	APC-750Fire (1:50)	Biolegend	302257	1:50
**Antibodies for flow cytometry – T-cell panel**				
**Target**	**Color**	**Supplier**	**Cat. Nr.**	**μl/staining**
AnnV	AF350	ThermoFisher	A23202	1
CD56	BUV395	BD	740299	3

CD28	BUV496	BD	741168	2
CD183 CXCR3	BUV563	BD	741406	7
CD80	BUV615	BD	751209	3.5
CD366 TIM3	BUV737	BD	748820	3
CD8	BUV805	BD	612889	1.2
CD152 CTLA4	BV421	BioLegend	369606	3.5
CD223 LAG3	BV480	BD	746609	1
HLA-DR	BV570	BioLegend	307637	1
CD134 Ox40	BV605	BD	745217	1
CD69	BV650	BioLegend	310933	1
TIGIT	BV711	BD	747839	1
CD279 PD-1	BV750	BioLegend	329965	1
CD197 CCR7	BV786	BD	566759	2.5
CD27	VioBright FITC	Miltenyi	130-114-160	0.5
CD3	Sparkblue 550	BioLegend	344851	1
CD95	BB700	BD	566543	1
γδTCR	PerCPeF710	ThermoFisher	46-9959-42	1.25
CD137 41BB	PE	Miltenyi	130-119-885	7
CD4	cFluorYG584	Cytek	R7-20041	0.5
CD154 CD40L	PE-Dazzle594	BioLegend	310840	1
CD244	PE-Cy5.5	ThermoFisher	35-5838-42	1
CD25	eFluor450	Life technologies	48-0257-42	2.5
CD160	PE-Cy7	BioLegend	341211	5
CD57	APC	BioLegend	359610	2
CD45RA	Spark NIR 685	BioLegend	304168	2
CD127	APC-R700	BD	565185	3
CD38	APC-Fire810	BioLegend	303550	1
Brilliant Stain Buffer Plus		BD	566385	10
True stain monocyte blocker		BioLegend	426103	5

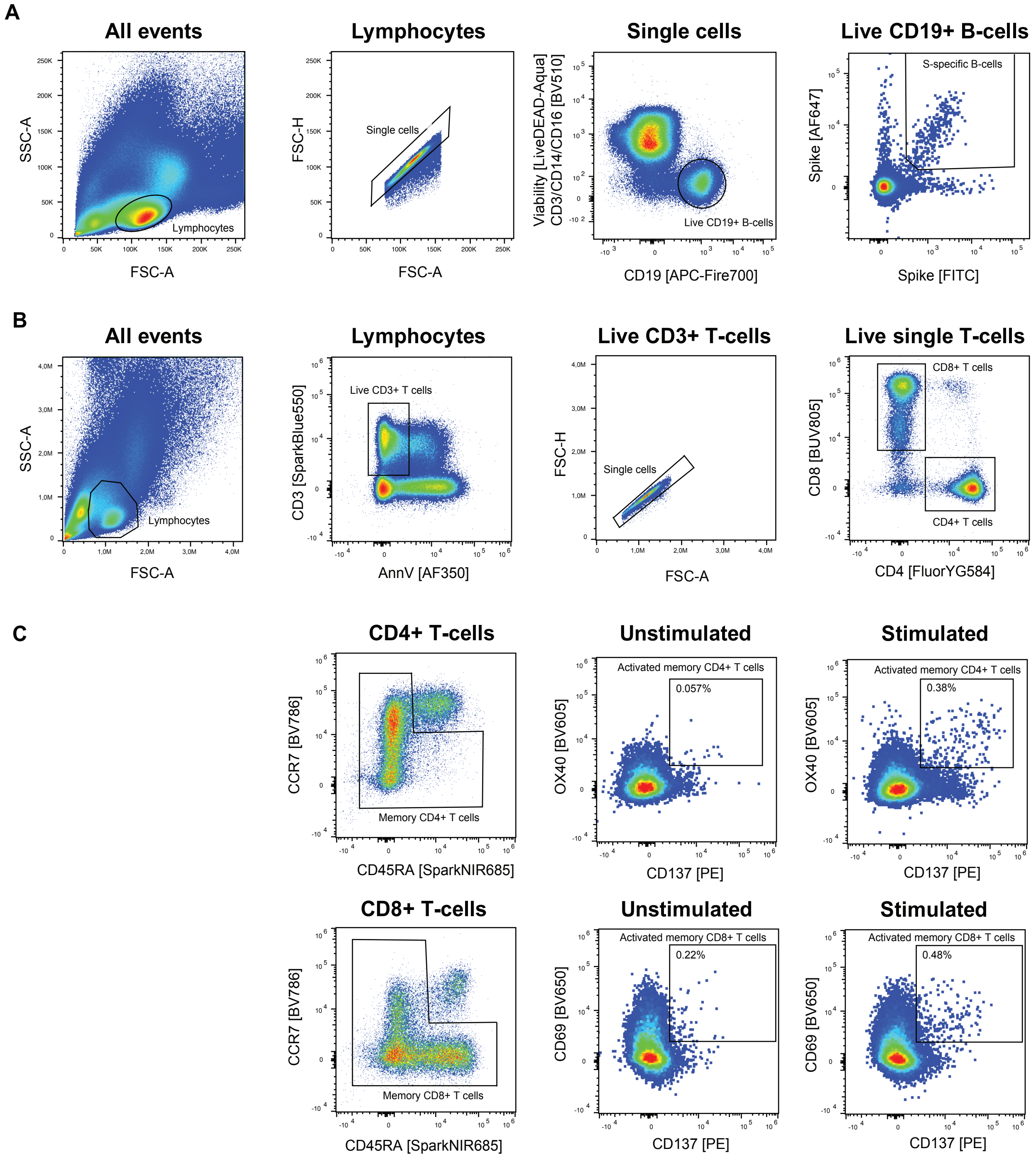

Supplemental Figure 1. Classical gating strategy of S-specific B-cells, memory CD4+ and CD8+ T-cells. **A**) S-specific B-cells were gated as; Lymphocytes [FSC-A, SSC-A] > singlets [FSC-A/FSC-H] > viable [Aqua-LiveDEAD]– and CD3– [BV510], CD14– [BV510], CD16– [BV510] and CD19+ [APCFire700] > Spike double-positive events [FITC]/[AF647] **B**) CD4+ and CD8+ T-cells were gated as; Lymphocytes [FSC-A, SSC-A] > CD3+ [Sparkblue550] and viable [AnnexinV-AF350] > CD4+ [FluorYG584] or CD8+ [BUV805], respectively. **C**) Memory CD4+ and CD8+ T-cells were gated as; CD4+ or CD8+T-cells (panel B) > exclude double-positive CCR7 [BV768] and CD45RA [SparkNIR685] events. S-specific memory CD4+ T-cells were gated as; CD137+ [PE] and OX40+ [BV605]. S-specific memory CD8+ T-cells were gated as; CD137+ [PE] and CD69+ [BV650]. The cut-off for S-peptide-specific CD4+ and CD8+ T-cells was determined using carrier-stimulated controls.

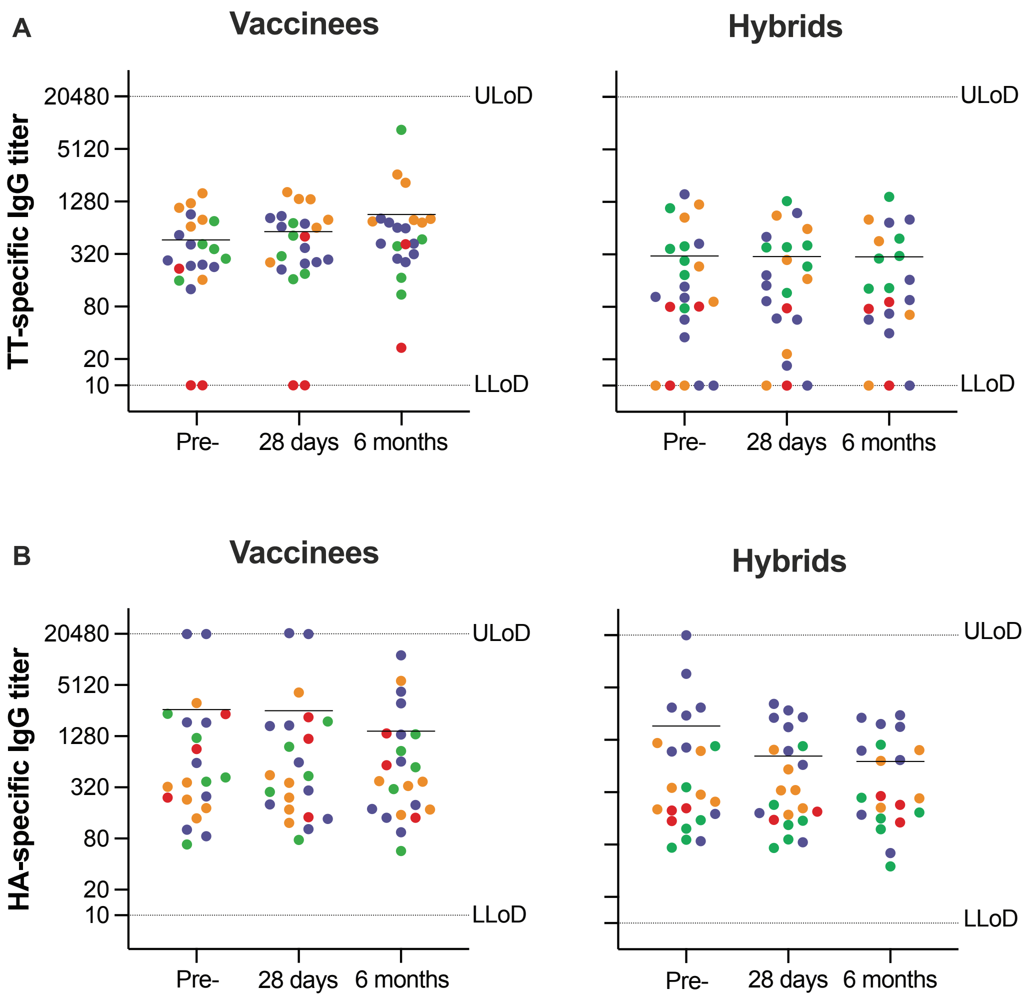

Supplementary figure 2. A) Serum Tetanus Toxoid (TT)-specific IgG titers and **B**) 2009 Influenza hemagglutinin (HA)-specific IgG titers of vaccinees and hybrid immune individuals pre-vaccination, 28 days post-vaccination and 6 months post-vaccination. Individual values are color coded per group consistant with all other graphs. Dotted lines represent the upper (ULoD) and lower limit of detection (LLoD). No significant differences were found by multiple comparison test with Mixed-effects analysis for paired data.

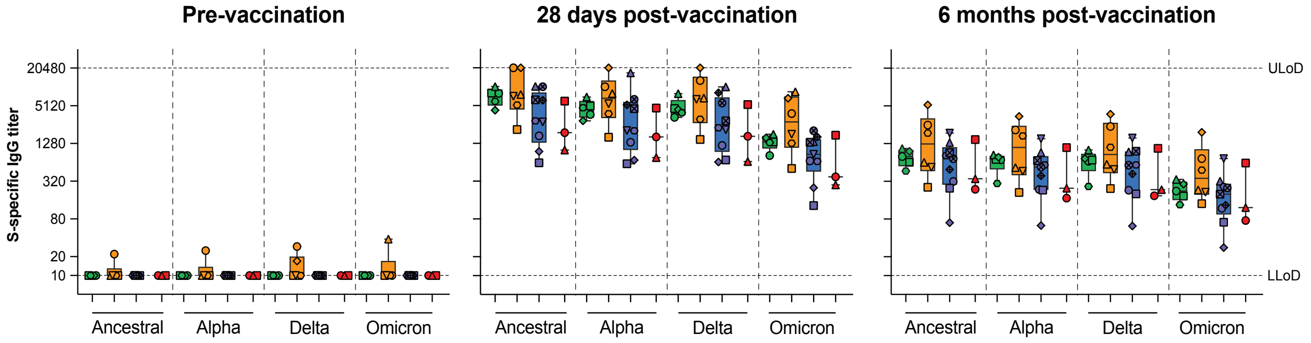

Supplemental Figure 3. Serum SARS-CoV-2 S-specific binding IgG titers to the ancestral, alpha, delta and omicron variants in controls, CKD, Dialysis and KTR vaccinees. Central line, box and whiskers represent the mean, interquartile range and minimum and maximum values respectively. Dotted lines represent the upper (ULoD) and lower limit of detection (LLoD). No statistical differences were found according to Mann Whitney-U test.

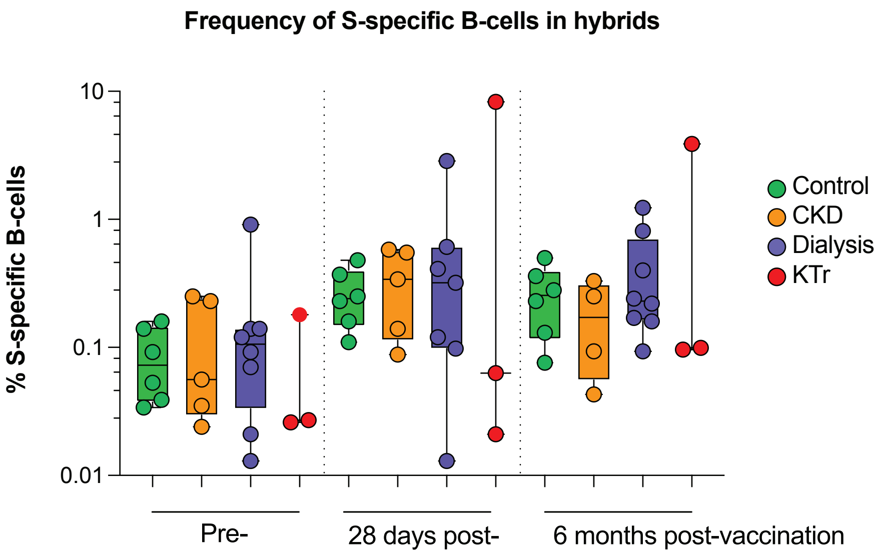

Supplemental Figure 4. Percentage of S-specific B-cells in hybrid immune individuals does not differ between groups at the different timepoints as assessed by Mann-Whitney-U test. Central line, box and whiskers represent the mean, interquartile range and minimum and maximum values respectively.

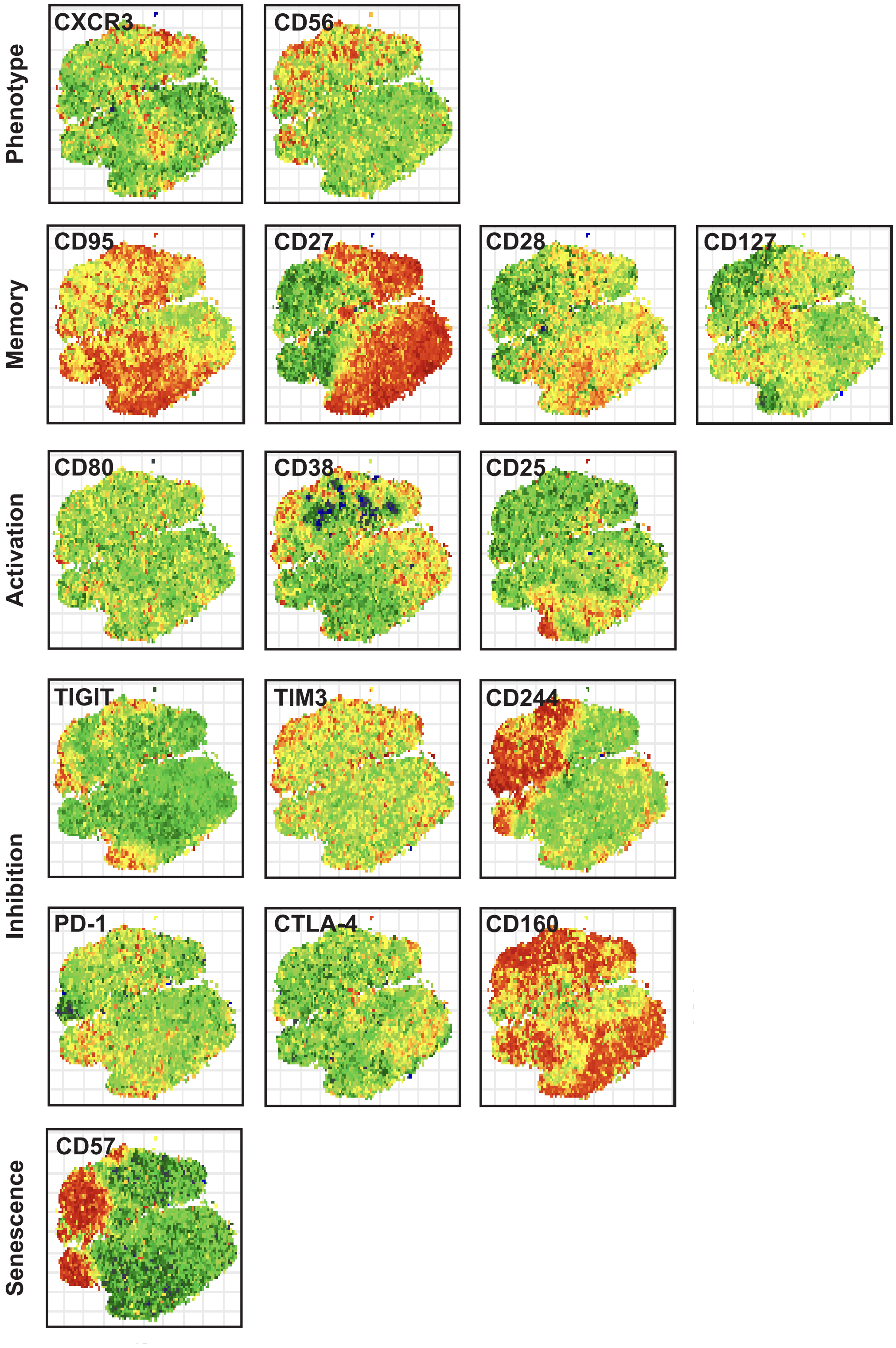

Supplemental Figure 5. Expression of phenotypic, memory, activation, inhibition and senescence markers in the t-SNE as determined by the mean fluorescent intensity using spectral flow cytometry. Expression of CD4, CD8, TCRyd, CD45RA and CCR7 is shown in Figure 4C. Markers for antigen-specific activation (OX40, CD137 and CD69) were not included in generating the tSNE map but instead used to identify S-specific CD4+ and CD8+ T-cells in relation to T-cells with similar phenotypes but different specificity.

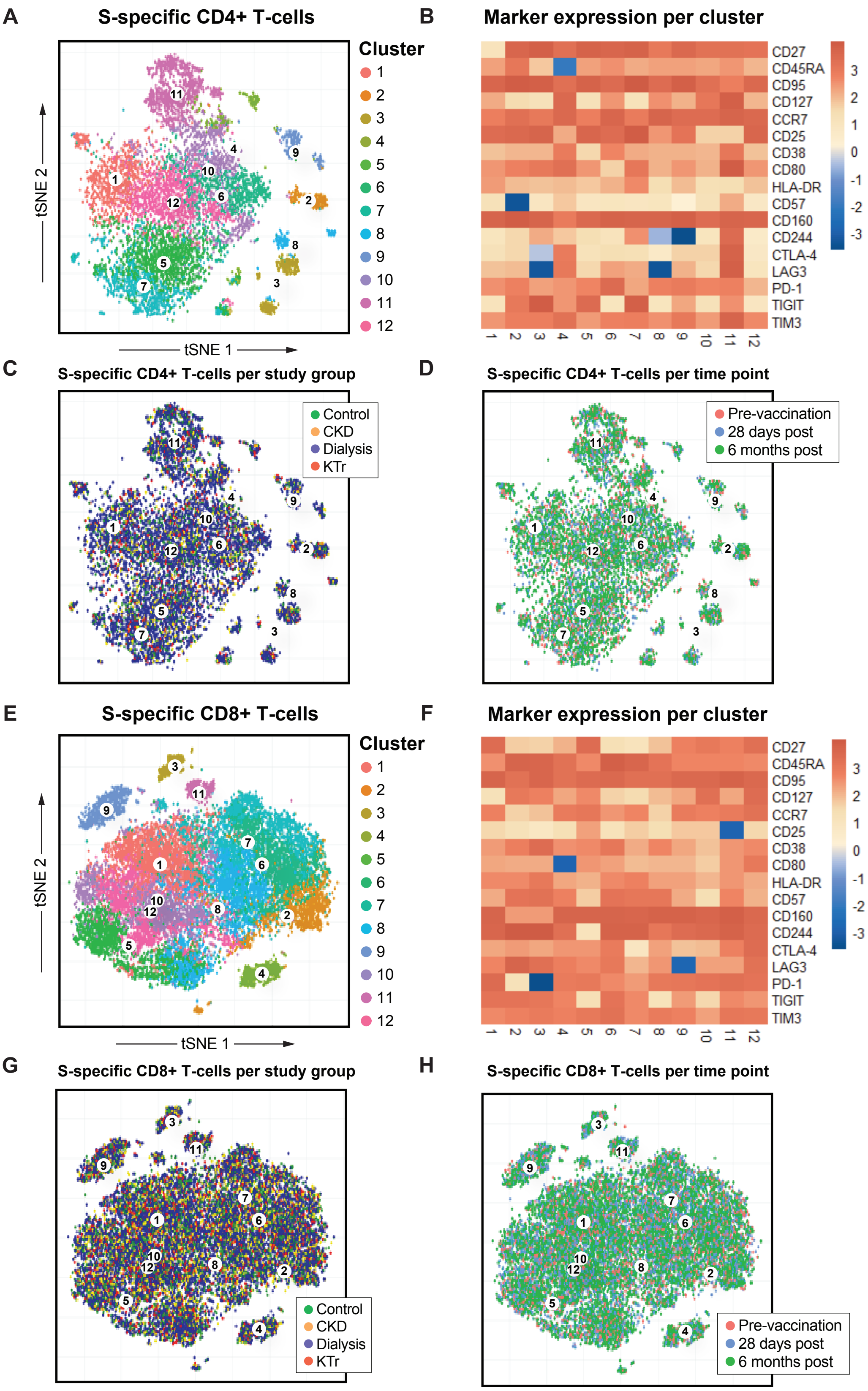

Supplemental Figure 6. Longitudinal phenotypic analysis of S-specific T-cells in kidney disease patients and controls. **A**) Population cluster identification in high-dimensional 29-color flow cytometry data using t-distributed stochastic neighbor embedding and projection (t-SNE) dimensionality reduction. Visualization is performed on combined pre-vaccination, 28 days and 6 months post second vaccination stimulated CD4+CD137+OX40+ T-cells of controls, CKD, dialysis and KTR participants. **B**) Heatmap of the expression profile of activation, inhibition and senescence markers identified using unsupervised cluster analysis. **C**) t-SNE plot of CD4+CD137+OX40+ T-cells color coded for controls, CKD, dialysis and KTR participants and **D**) color coded for pre-vaccination, 28 days and 6 months post second vaccination. **E**-**H**) Similar analysis shown in panel A-D for CD8+CD69+CD137+ T-cells.

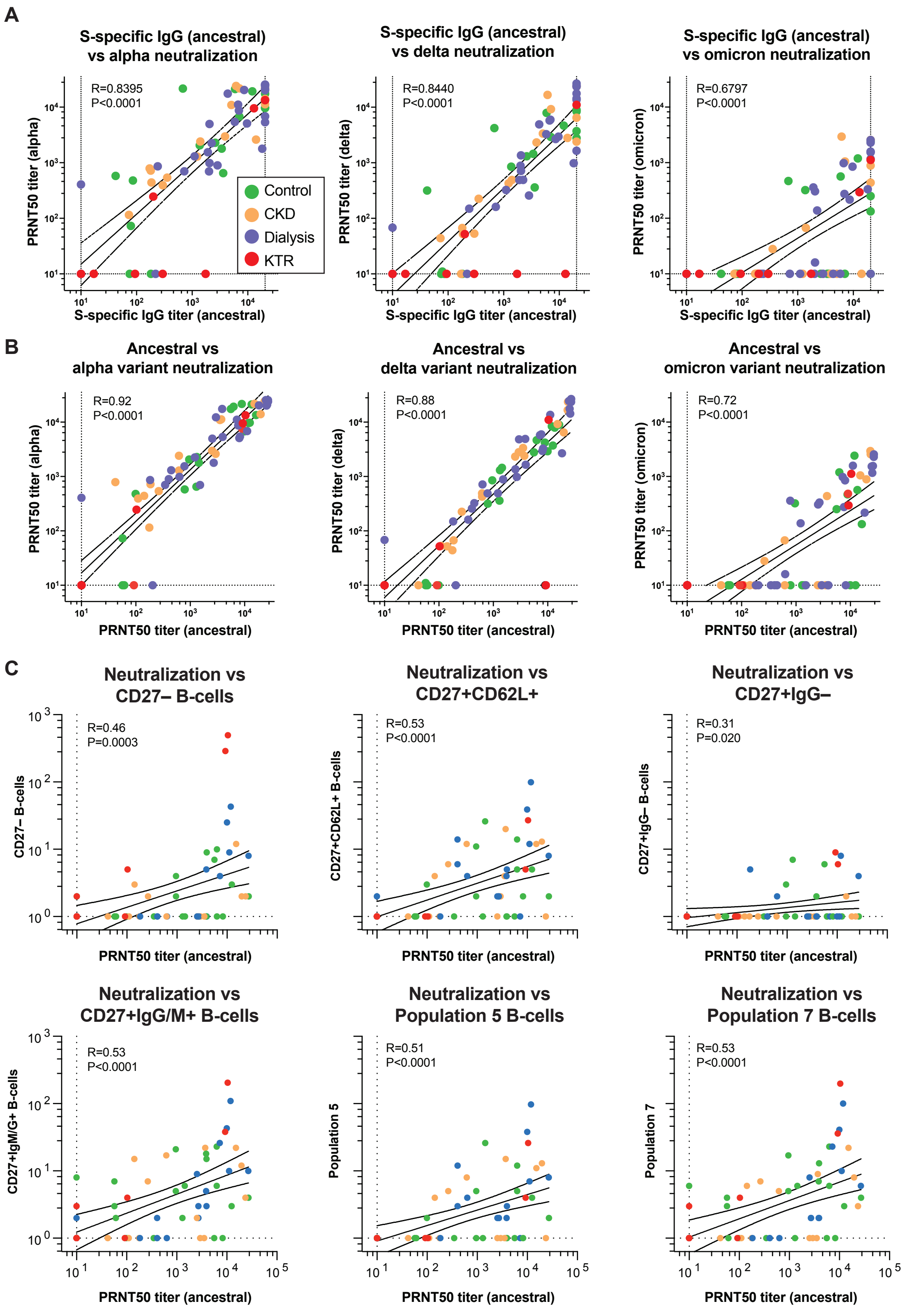

Supplemental Figure 7. A) Serum S-specific IgG for the alpha, delta and omicron variants correlate with S-specific binging IgG for the ancestral variant. **B**) Serum alpha, delta and omicron variant neutralizing antibodies correlate with ancestral neutralizing antibodies. **C**) The frequency of S-specific B-cells of the phenotypes defined in Figure 3A/B correlate with serum ancestral variant neutralizing antibodies. Linear regression was performed on ^{10}log-transformed data. Spearman rank correlation was used to calculate R and significance. Regression and 95% confidence intervals are plotted. Horizontal and vertical dotted lines depict the LLoD and ULoD.
