# Supplementary material for: SARS-CoV-2-specific immune responses converge in kidney disease patients and controls with hybrid immunity
Source: NPJ Vaccines. 2024 May 28;9:93. doi: 10.1038/s41541-024-00886-0 (PMC11133345; doi:10.1038/s41541-024-00886-0)
Supplement: Supplementary file 2 — REPORTING SUMMARY [file 41541_2024_886_MOESM2_ESM.pdf]

nature portfolio

Corresponding author(s): Rory D. de Vries and Gijsbert P. van Nierop

Last updated by author(s): 20/02/2024

Reporting Summary

Nature Portfolio wishes to improve the reproducibility of the work that we publish. This form provides structure for consistency and transparency in reporting. For further information on Nature Portfolio policies, see our Editorial Policies and the Editorial Policy Checklist.

Statistics

For all statistical analyses, confirm that the following items are present in the figure legend, table legend, main text, or Methods section.

n/a	Confirmed	
☐	☒	The exact sample size (n) for each experimental group/condition, given as a discrete number and unit of measurement
☒	☐	A statement on whether measurements were taken from distinct samples or whether the same sample was measured repeatedly
☐	☒	The statistical test(s) used AND whether they are one- or two-sided<br>*Only common tests should be described solely by name; describe more complex techniques in the Methods section.*
☐	☒	A description of all covariates tested
☐	☒	A description of any assumptions or corrections, such as tests of normality and adjustment for multiple comparisons
☐	☒	A full description of the statistical parameters including central tendency (e.g. means) or other basic estimates (e.g. regression coefficient) AND variation (e.g. standard deviation) or associated estimates of uncertainty (e.g. confidence intervals)
☒	☐	For null hypothesis testing, the test statistic (e.g. F, t, r) with confidence intervals, effect sizes, degrees of freedom and P value noted<br>*Give P values as exact values whenever suitable.*
☒	☐	For Bayesian analysis, information on the choice of priors and Markov chain Monte Carlo settings
☐	☒	For hierarchical and complex designs, identification of the appropriate level for tests and full reporting of outcomes
☒	☐	Estimates of effect sizes (e.g. Cohen's d, Pearson's r), indicating how they were calculated

Our web collection on statistics for biologists contains articles on many of the points above.

Software and code

Policy information about availability of computer code

Data collection	Data was collected with BD FACS Diva software for BD Fortessa and SpectroFlo software for Cytek Aurora.
Data analysis	Statistical analyses were carried out with GraphPad Prism software version 9.1.2 unsupervised analysis were carried out with FlowJo software v.10. , FlowSOM algorithm (v.2.5.2) and Rstudio software version 4.2.1 packages CytoTree (v.1.6.0) and FlowSOM (v.2.5.2).

For manuscripts utilizing custom algorithms or software that are central to the research but not yet described in published literature, software must be made available to editors and reviewers. We strongly encourage code deposition in a community repository (e.g. GitHub). See the Nature Portfolio guidelines for submitting code & software for further information.

Data

Policy information about availability of data

All manuscripts must include a data availability statement. This statement should provide the following information, where applicable:

- Accession codes, unique identifiers, or web links for publicly available datasets
- A description of any restrictions on data availability
- For clinical datasets or third party data, please ensure that the statement adheres to our policy

The data and R code that support the findings of this study are available from the corresponding author upon reasonable request.

Human research participants

Policy information about studies involving human research participants and Sex and Gender in Research.

Reporting on sex and gender	Findings in this study only apply to sex (biological trait) that were self-reported. We did not include sex and gender analysis as it was not relevant to this study.
Population characteristics	Four different cohorts were included; cohort A: participants without kidney disease (eGFR >45 mL/min/1.73m2); cohort B: patients with CKD stage G4/5 (eGFR <30 mL/min/1.73m2); cohort C: patients undergoing hemo- or peritoneal dialysis; and cohort D: Kidney transplant recipients.
Recruitment	From all participants who participated in the RECOVAC IR study we measured humoral and cellular immune responses of the hybrid individuals (baseline Anti-S1 antibody levels >10 BAU/mL)
Ethics oversight	The Dutch Central Committee on Research Involving Human Subjects (CCMO, NL76215.042.21) and the institutional review board of the Erasmus MC Rotterdam (MEC2020-662) approved the study

Note that full information on the approval of the study protocol must also be provided in the manuscript.

Field-specific reporting

Please select the one below that is the best fit for your research. If you are not sure, read the appropriate sections before making your selection.

☒ Life sciences ☐ Behavioural & social sciences ☐ Ecological, evolutionary & environmental sciences

For a reference copy of the document with all sections, see nature.com/documents/nr-reporting-summary-flat.pdf

Life sciences study design

All studies must disclose on these points even when the disclosure is negative.

Sample size	No sample-size calculation was performed, sample size was established by including the hybrid participants of the RECOVAC IR study (n=24) and RECOVAC IR participants who were aged and sex matched to the hybrid participants (n=24).
Data exclusions	There was no data excluded during this study from the analyses.
Replication	All serological assays were performed in duplicates. For FACS analysis all were performed once due to a limited amount of cells available.
Randomization	Allocation was not random because we were intrested in the SARS-CoV-2 specific repsonses in the different patient groups.
Blinding	Blinding was not relevant in our study because all participants received mRNA-1273 vaccination.

Reporting for specific materials, systems and methods

We require information from authors about some types of materials, experimental systems and methods used in many studies. Here, indicate whether each material, system or method listed is relevant to your study. If you are not sure if a list item applies to your research, read the appropriate section before selecting a response.

Materials & experimental systems

n/a	Involved in the study	
☐	☒	Antibodies
☐	☒	Eukaryotic cell lines
☒	☐	Palaeontology and archaeology
☒	☐	Animals and other organisms
☒	☐	Clinical data
☒	☐	Dual use research of concern

Methods

n/a	Involved in the study	
☒	☐	ChIP-seq
☐	☒	Flow cytometry
☒	☐	MRI-based neuroimaging

Antibodies

Antibodies used	Details regarding the commercial antibodies utilized, including information on companies, catalog numbers, and clones, are furnished in both the materials and methods section and Supplementary Table.
Validation	All primary and secondary antibodies used in serological assays and flowcytometry were validated to be used for this assays corresponding manufacturers. All the antibodies were used following the guidlines of the manufacturers.

Eukaryotic cell lines

Policy information about cell lines and Sex and Gender in Research

Cell line source(s)	The Calu-3 cells are originally from ATCC
Authentication	As confirmed by ATCC
Mycoplasma contamination	The cells are periodically tested for mycoplasma contamination. If they are negative, we use them for experiments.
Commonly misidentified lines (See ICLAC register)	x

Flow Cytometry

Plots

Confirm that:

☒ The axis labels state the marker and fluorochrome used (e.g. CD4-FITC).

☒ The axis scales are clearly visible. Include numbers along axes only for bottom left plot of group (a 'group' is an analysis of identical markers).

☒ All plots are contour plots with outliers or pseudocolor plots.

☒ A numerical value for number of cells or percentage (with statistics) is provided.

Methodology

Sample preparation	Blood samples were obtained from participants enrolled in the hybrid and vaccines RECOVAC IR study at baseline, 28 days, and 6 months post two doses of mRNA-1273 vaccine. Peripheral blood mononuclear cells (PBMCs) were isolated via Ficoll and promptly frozen for later use. Upon thawing, the samples underwent antibody staining.
Instrument	BD FACS Fortessa and 5-laser Cytek Biosciences Aurora spectral flow cytometer
Software	FlowJo software v.10. , FlowSOM algorithm (v.2.5.2) and Rstudio software version 4.2.1
Cell population abundance	N/A
Gating strategy	B and T cells were initially gated based on size, singlets, and dead cell staining. For B-cells, those expressing CD19+ and negative for CD3-, CD14-, and CD16- were selected. For T-cells, CD3+ cells were first isolated and subsequently separated into CD4+ and CD8+ populations. Further separation involved categorizing CD4+ T-cells as CD137+OX40+ and CD8+ T-cells as CD137+CD69+.

☒ Tick this box to confirm that a figure exemplifying the gating strategy is provided in the Supplementary Information.
